# Spurious Hyperkalemia Without Hemolysis: A Case-Based Presentation of Preanalytical Errors and Diagnostic Pitfalls

**DOI:** 10.3390/diagnostics16162676

**Published:** 2026-08-21

**Authors:** Alina Umińska, Ewa Wieczorek-Breitzke, Agnieszka Ćwiklińska

**Affiliations:** 1 Analytical Laboratory, Dr. Władysław Biegański Regional Specialist Hospital, 86-300 Grudziądz, Poland; 2 Department of Clinical Chemistry, Medical University of Gdańsk, 80-211 Gdańsk, Poland

**Keywords:** potassium, preanalytical error, preanalytical phase, spurious hyperkalemia

## Abstract

**Background and Clinical significance:** Potassium is one of the most frequently measured laboratory parameters used to assess a patient’s clinical status and is also one of the analytes most commonly affected by preanalytical errors. **Case Presentation:** A potassium test was ordered for a 63-year-old male patient. The result obtained was 6.4 mmol/L, indicating hyperkalemia, and a medical consultation was recommended in the laboratory report. As the potassium result was inconsistent with the patient’s clinical status, a new blood sample was analyzed the next day. This yielded a result that was 1.3 mmol/L (20%) lower. A detailed interview with the patient and the staff responsible for blood sample collection revealed that the spurious hyperkalemia was most likely caused by prolonged storage of the blood sample in a refrigerator implemented because of high ambient temperatures. Further analysis demonstrated that after storage of blood samples at low temperature, potassium concentration increased significantly on average by 5% and 20% at the 4 h and 8 h time points, respectively. **Conclusions:** Low temperature and prolonged blood sample storage before analysis significantly affect potassium results. Since even a slight alteration in potassium levels can have a severe impact on a patient’s health and may require immediate action, obtaining accurate potassium results requires ensuring appropriate conditions for sample storage and transport, and proper sample handling. This is particularly important in laboratories serving large geographical areas, where prolonged storage and transportation of blood samples can considerably extend the preanalytical phase.

## 1. Introduction

Potassium is a crucial electrolyte for proper cell function in the human body and is one of the most frequently measured laboratory parameters used to assess a patient’s clinical status [1]. This cation plays a key role in maintaining the osmotic balance of the cells, regulating the osmotic pressure of body fluids alongside sodium, and contributing to acid-base homeostasis. Most of the potassium in the body is located in the intracellular space, with only ~1% present in the extracellular environment [1]. The reference serum potassium interval is usually defined as the range between 3.5 mmol/L and 5.0 mmol/L [2]. 

Excessively high or low potassium concentrations have a very negative impact on the patient’s condition. They affect neuromuscular conduction, gastrointestinal function, and, most importantly, cardiac activity, leading to rhythm disturbances and impaired intraventricular conduction, which may result in myocardial infarction [1,2]. Hypokalemia can result from insufficient dietary intake, excessive gastrointestinal or urinary loss, or a shift of potassium from the extracellular to the intracellular space during alkalosis, or due to the action of hormones such as insulin, aldosterone, catecholamines or thyroid hormones. The primary symptoms of hypokalemia are muscle weakness affecting skeletal muscles (reduced muscle strength), smooth muscles (gastrointestinal atony), and cardiac muscle (arrhythmia) [1,2]. Hyperkalemia is most commonly caused by excessive dietary intake, infusion fluids, medications or transfusion of hemolyzed blood, as well as by reduced renal excretion. It may also result from the potassium release due to cell breakdown, or from a shift of potassium from the intracellular to the extracellular space during acidosis. Hyperkalemia mainly manifests through disturbances in cardiac muscle function, including bradycardia and conduction abnormalities [1,2]. Disorders of potassium homeostasis require immediate treatment, which must be tailored to the severity of the imbalance and any comorbid conditions. Therefore, accurate, rapid, and error-free potassium measurement is crucial [3]. 

In medical laboratories, potassium concentration is routinely determined in extracellular fluid (either serum or plasma, with lithium heparin), using ion-selective electrode (ISE) methodology. Although ISE methods have some limitations, they are considered a reliable means of determining potassium concentration, and the most common source of error in potassium determination is considered the preanalytical phase [3,4]. In this paper, we present a case-based study in which the storage conditions of blood samples resulted in spurious hyperkalemia.

## 2. Case Presentation

A 63-year-old man in very good health and with no signs of illness visited the general practice (GP) office for a check-up visit at his wife’s urging. Control laboratory tests were ordered, including a test to measure potassium concentration. 

The blood sample was collected at the GP office in the early morning hours on 25 June (Table 1). The next morning, the patient’s wife, who worked as a nurse at the GP office, received the laboratory result indicating an increased potassium level of 6.4 mmol/L, along with a comment from the laboratory recommending medical consultation (Table 1). The patient had no history of kidney disease or any other disorders known to cause hyperkalemia. He was not receiving any medications known to affect potassium level and he also denied potassium supplementation. Despite being in good clinical condition and exhibiting no signs or symptoms of hyperkalemia, he took time off work before noon and came to the GP office, where blood was taken again for potassium determination. The result obtained that day was 1.3 mmol/L (20%) lower compared to the previous day’s result (Table 1). 

To investigate the reason for the first high potassium result, an interview was conducted concerning the patient preparation and sample handling at the GP office. The patient had no forced physical activity prior to blood collection and there was no pumping of the fist or muscle contraction during the procedure. The tourniquet application was not prolonged. Contamination of the sample with potassium due to the incorrect order of draws, or contamination with hand hygiene products, has been ruled out. Traumatic venipuncture and hemolysis have also been ruled out, as all samples were assessed for the HIL index, a quality control measure used to detect hemolysis, icterus, and lipemia. However, attention was drawn to the long period of time between blood collection and obtaining the result from the laboratory, suggesting a very long time between sample collection and analysis. Indeed, it turned out that all the samples had been collected from the GP office by the courier at around 1 p.m. (Table 1). Furthermore, the phlebotomist who collected the blood revealed that, due to the high ambient temperature at this time of the year, all blood samples at the GP office were stored in the refrigerator until they were collected by the courier. In summary, two preanalytical variables differed between the two potassium measurements in our patient: storage temperature and storage duration, both of which could have affected the obtained results.

To assess whether blood storage conditions could have contributed to the observed spurious hyperkalemia, we performed an experiment in which the potassium level was determined in the samples as soon as possible after blood collection (within 30 min) (“0 h” time point), and after four hours (“4 h” time point), and after eight hours (“8 h” time point) of whole blood sample storage at room temperature or in the refrigerator. Specifically, we used anonymized blood samples of thirty-four patients—the back-up samples that had not been used in routine laboratory analyses and were intended for disposal. Samples were collected into commercially available tubes with clotting activator (S-Monovette^®^ Serum CAT, Sarstedt AG & Co. KG, Nümbrecht, Germany), and unseparated whole blood samples were stored under different experimental conditions. Blood samples from seventeen patients were stored at room temperature, while samples from the remaining seventeen patients were refrigerated. The temperature in the areas where the samples were stored was monitored and maintained at 20 °C and 5 °C, respectively. After a defined storage period, the samples were centrifuged at 2200× *g* for 5 min at room temperature, and the potassium level in the serum was measured without delay using the indirect ISE method on an Attelica Solution CH930 analyzer (Siemens Healthineers AG, Erlangen, Germany). The HIL index was automatically determined by an analyzer for all serum samples, and no hemolysis, lipemia or icterus that could affect the results was detected. Specifically, all samples had a hemolysis index of 0, an icterus index ≤ 1, and a lipemia index of 0. These values were below the manufacturer-defined values for clinically significant interference with potassium measurement (alert index: a hemolysis index of 1, an icterus index of 6, and a lipemia index of 6).

**Figure 1:**
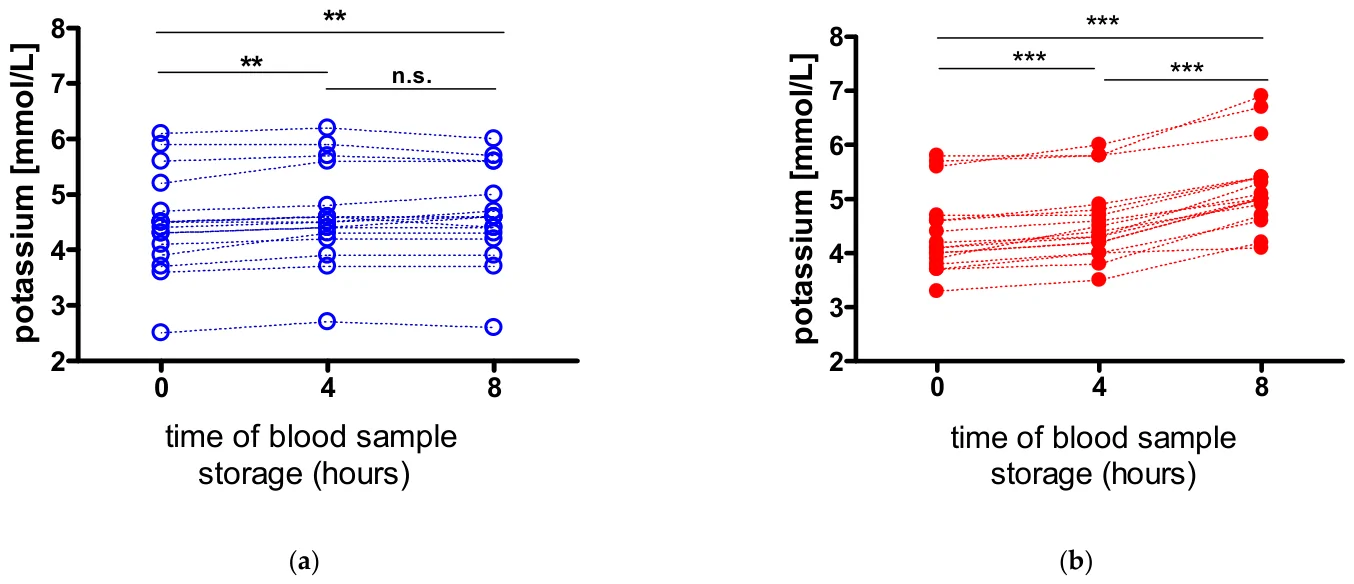
Serum potassium concentration (mmol/L) after storage of whole blood samples for four and eight hours at room temperature (**a**) and under refrigerated conditions (**b**). Data were analyzed using Repeated Measures ANOVA, followed by Bonferroni’s Multiple Comparison Test as a post hoc analysis; *** *p* < 0.001, ** *p* < 0.01, n.s. *p* > 0.05. Total n = 34 (samples stored at room temperature, n = 17; and samples stored in a refrigerator, n = 17).

For samples stored at room temperature, the potassium level was 4.5 ± 0.9 mmol/L (mean ± standard deviation) at the “0 h” time point, and 4.6 ± 0.9 mmol/L (Δ + 3.2%; 95%CI: 1.9–4.6%), and 4.6 ± 0.8 mmol/L (Δ + 3.1%; 95%CI: 1.3–5.0%) at the “4 h”, and “8 h” time points, respectively (η^2^ = 0.387). For samples stored in a refrigerator, the potassium concentration was 4.4 ± 0.7 mmol/L at the “0 h” time point. Following storage, the potassium concentration increased significantly (*p* < 0.001), reaching 4.6 ± 0.7 mmol/L (Δ + 5.0%; 95%CI: 3.2–6.8%), and 5.2 ± 0.8 mmol/L (Δ + 19.8%; 95%CI: 16.6–23.1%) at the “4 h”, and “8 h” time points, respectively (η^2^ = 0.904) (Figure 1).

## 3. Discussion

Potassium is highly sensitive to various types of preanalytical errors [3]. Erroneous potassium results are most commonly caused by excessive leakage of potassium from cells resulting from hemolysis of blood cells. However, there can also be other reasons for spurious potassium results that are not related to hemolysis, including factors related to the patient, blood collection procedure, specimen type, and specimen processing [3].

One of the important preanalytical causes of spurious hyperkalemia is inappropriate blood sample storage conditions. Thus, current Clinical & Laboratory Standards Institute (CLSI) guidelines recommend storing blood samples for potassium determinations at room temperature (15–24 °C) and centrifuging the samples as quickly as possible. The stability of potassium in uncentrifuged blood samples stored at 2–8 °C is less than 2 h, and for samples stored at room temperature, it can be as long as 4 h. Centrifugation and physical separation of serum from blood cells allow for extending the stability of the sample for potassium determination up to 24 h for both temperatures mentioned above [5].

The effect of blood sample storage conditions on potassium results is largely associated with the impact of storage temperature on the sodium-potassium pump (Na^+^/K^+^ pump) activity, which alters potassium distribution between the intracellular and extracellular compartments [3]. Namely, low temperature inhibits the activity of the Na^+^/K^+^ pump in vitro, resulting in potassium leaking out of cells and increasing its level in the serum [6].

**Figure 2:**
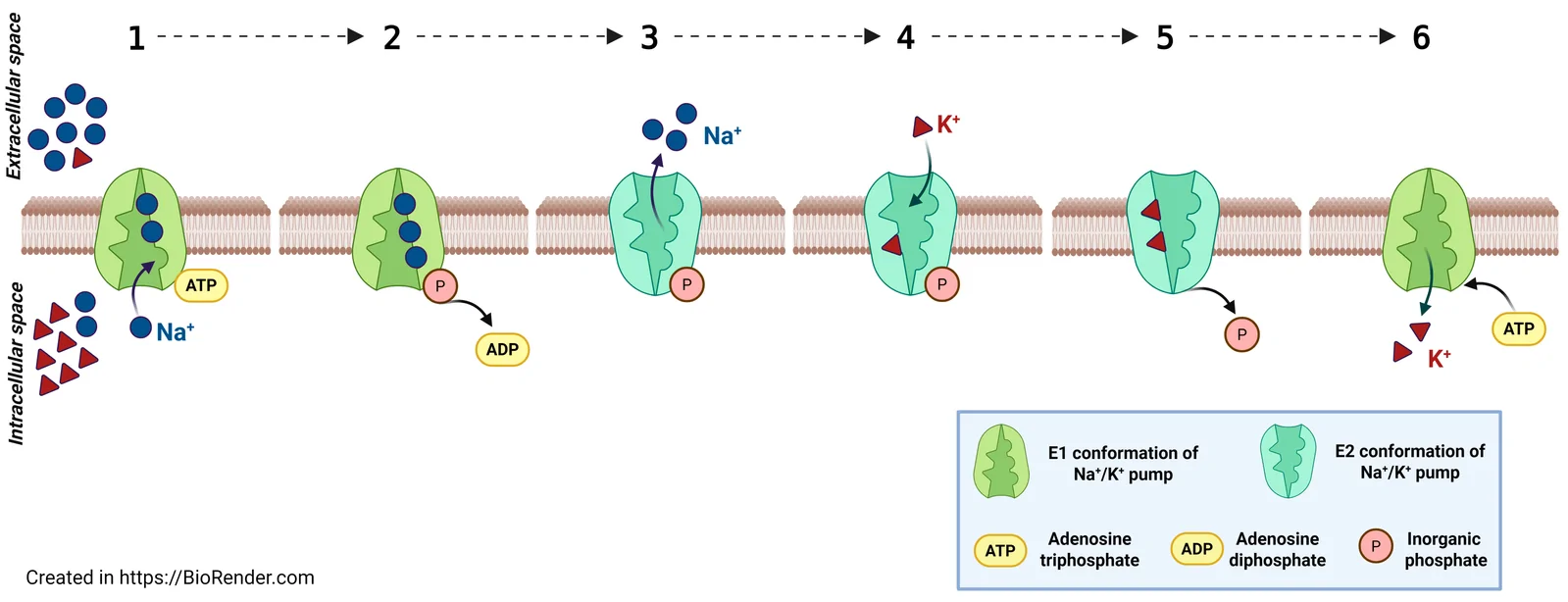
Mechanism of action of the Na^+^/K^+^ pump.

The Na^+^/K^+^ pump, also known as Na^+^/K^+^-ATPase, is a transmembrane enzyme involved in the active transport of sodium and potassium ions across the cell membrane, against their concentration gradients. It is found in the lipid bilayer of cellular membranes throughout the body, including in blood cells [7]. Under physiological conditions, one ATP molecule and three sodium ions bind to the Na^+^/K^+^-ATPase on the intracellular side. ATP then undergoes hydrolysis, which leads to the phosphorylation of the pump and a conformational change from the E1 position (open towards the cell interior) to the E2 position (open towards the extracellular environment) (Figure 2). This results in the translocation of three sodium ions from inside the cell to the extracellular space. In the E2 position, the Na^+^/K^+^ pump exhibits a high affinity for potassium ions, exposing K^+^ binding sites, and binds two potassium ions from the extracellular space. This triggers dephosphorylation of the pump, causing it to return to the E1 conformation and release the K^+^ ions into the cell (Figure 2) [8].

The Na^+^/K^+^ pump is an enzyme whose proper function depends on several factors. These include the availability of ATP, as well as the appropriate concentrations of Na^+^, K^+^, and Mg^2+^, which act as cofactors. An optimal temperature is also required. The pump operates most efficiently at approximately 37 °C [9]. Studies have shown that reducing the environmental temperature at which the Na^+^/K^+^ pump functions significantly decreases its activity [9,10,11]. For most biological enzymes, a temperature reduction of 10 °C results in a 50% decrease in activity. This means that storing a whole blood sample at 4 °C substantially inhibits Na^+^/K^+^ pump activity [9]. Additionally, a reduced temperature impairs membrane fluidity, resulting in increased rigidity. This, in turn, negatively affects the ability of transmembrane proteins (including enzymes) to undergo conformational changes [12]. If the Na^+^/K^+^-ATPase pump does not function properly, sodium and potassium ions will move according to their respective concentration gradients: potassium ions leak out of blood cells into the plasma, while sodium ions will enter the cells. This results in apparent hyperkalemia and, to a lesser extent, apparent hyponatremia [7].

The effect of temperature on the Na^+^/K^+^ pump means that low temperatures are associated with pseudohyperkalemia, whereas high temperatures are associated with pseudohypokalemia. Interestingly, the impact of the ambient temperature on blood samples during transport and storage is responsible for the phenomena known as seasonal pseudohyperkalemia (observed in winter) and seasonal pseudohypokalemia (observed in summer) [13,14,15,16]. These phenomena particularly affect samples obtained from primary care and pose a significant challenge for laboratories serving large geographical areas, where the storage and transportation of blood samples can extend the preanalytical phase by up to several hours [14,15,17]. 

For instance, Afrin et al. showed that most critically elevated potassium results reported during winter in primary care were artefactual rather than indicative of true hyperkalemia, highlighting the significant impact of this issue on patient safety [18]. Also, a narrative review of literature in major scientific databases (PubMed, Scopus, Web of Science) using keywords relevant to the issue revealed cases in which the storage temperature-dependent spurious hyperkalemia led to severe consequences, including avoidable emergency referral and even admission to a hospital. The reported cases were mainly associated with familial pseudohyperkalemia, in which low temperatures promote potassium leakage from cells due to structural abnormalities and increased permeability of the erythrocyte membrane, but also with the seasonal pseudohyperkalemia phenomenon observed in winter (Table 2). 

In our patient, there was no history of hyperkalemia. Furthermore, the blood samples were collected in the summer and took at least six hours to be stored and transported to the laboratory. Therefore, given the high temperature and prolonged time of blood sample transportation, one would expect a falsely decreased potassium level, consistent with the phenomenon of seasonal pseudohypokalemia [25]. Conversely, in our case, a falsely elevated potassium level was observed and was likely related to storing the samples in a refrigerator, implemented because of high ambient temperatures. Furthermore, it emerged that all the samples collected at the GP office during this period of high ambient temperature were stored in the refrigerator, and increased potassium levels above the reference values were observed for many patients. A similar situation was described by Schlüter et al. Namely, the laboratory found a general elevation in potassium results of all samples in the summer, and upon establishing the possible cause of this phenomenon it, turned out that, due to long distances from GP offices (in which the samples were collected) to the laboratory, and to the high ambient temperatures, the laboratory had recently decided to implement transport at cold temperatures. Potassium level returned to normal after transport at ambient temperature was introduced [3]. This can indicate a lack of awareness of the adverse effects of storage conditions on potassium levels and highlights the need to educate medical staff regarding proper sample storage conditions. 

In our case, spurious hyperkalemia resulted in unnecessary distress for the patient and his wife, as well as unnecessary time off work, repeated testing (increasing analysis costs), and also unnecessary dietary restrictions imposed by the patient’s wife. However, a false potassium level can also have more serious consequences, such as an urgent unnecessary hospitalization or inappropriate therapies [21,22], and Furia et al. reported even the referral of the police force to find and help the patient with a very high false potassium result [26]. A potentially significant hypokalemia may also be masked [3]. Thayakaran et al. also identified an association between ambient temperature-related seasonal fluctuations in potassium levels and the prescribing activity of potassium-sensitive medications, including potassium supplements and angiotensin-converting enzyme (ACE) inhibitors [27]. These observations highlight the importance of storage and transport conditions on potassium levels which can be of special importance for patients with disorders such as familial pseudohyperkalemia, which additionally predisposes to temperature-dependent potassium leakage from cells [20,21,24,26]. 

A single-case study has some limitations [28]. Preanalytical case studies cannot represent the full variability of preanalytical errors across different patient populations, clinical settings, or specimen types. Case reports also do not allow assessment of prevalence or statistical significance, and their conclusions cannot be generalized beyond the specific circumstances described. Moreover, in our case, although the patient’s clinical condition was known and it did not suggest hyperkalemia, we did not have access to additional data such as renal function tests, complete blood count results, or electrocardiographic findings, which could more directly exclude other causes of hyperkalemia. On the other hand, preanalytical single-case examples remain valuable in laboratory medicine because they illustrate real-world failures in preanalytical processes, highlight vulnerabilities in routine workflows, and provide concrete evidence of how deviations from recommended transport and storage conditions can compromise specimen integrity. Moreover, to address some of the limitations of our single-case observation, we performed a validation study using 34 anonymized samples and the mean increase in potassium level after prolonged blood sample storage under refrigerated conditions was comparable to that observed for the patient, approximately 20%. However, individual results varied from 6% to 31%, indicating substantial interindividual variability that has also been observed by others [21,22,23]. This variability may be related to various factors, including, inter alia, subtle differences in sample collection and handling, differences in sample-specific cellular composition and intracellular potassium content, as well as pre-existing hereditary or acquired conditions affecting potassium release from cells, such as rare genetic conditions associated with increased temperature-dependent erythrocyte leakage and other conditions predisposing to spurious potassium results, including thrombocytosis and severe leukocytosis [3]. Despite the interindividual differences observed in our study and other reports, they all indicate that the impact of storage conditions on potassium measurements is of great importance. Furthermore, several studies demonstrated that this phenomenon affects the general population and may lead to changes in patient management, as well as increased healthcare utilization and higher healthcare costs [13,14,18,27].

## 4. Conclusions

Low temperature and prolonged blood sample storage before analysis can significantly affect potassium results. Since even a slight alteration in potassium level can severely impact a patient’s health and may require immediate action, accurate potassium results require ensuring appropriate storage, transport, and handling of blood samples. This is particularly important in laboratories serving large geographical areas, where the time of storage and transportation of blood samples can considerably extend the preanalytical phase. Moreover, this issue may become increasingly relevant for laboratory quality management as periods with extreme summer temperatures become more frequent, potentially encouraging healthcare professionals to store blood samples under refrigerated conditions, with the intention of preserving sample quality. On the other hand, prolonged exposure of blood samples to high ambient temperatures may lead to pseudohypokalemia. These observations emphasize the importance of ensuring appropriate sample transport, storage and handling conditions.

The following measures may be considered:

### 4.1. Training and Education

Educational activities for personnel responsible for sample collection, as well as for staff overseeing the conditions and timing of sample transport and storage. These activities should include regular training sessions, as well as the provision of instructions and procedures at workstations.

### 4.2. Operational Actions

The reduction in transport time and ensuring that samples are stored and transported in the appropriate conditions, paying particular attention to maintaining an optimal temperature, and avoiding temperature fluctuations. 

If it is not possible to shorten transport time or optimize conditions, the following can be considered:

- Using tubes with separation gel and distributing centrifuges to sample collection points to allow for sample centrifugation and serum separation prior to transportation. Turner et al. demonstrated that introducing centrifuges to GP offices and centrifuing samples after collection significantly reduced the effect of seasonal variation in serum potassium levels [17].
- Delivering samples to the nearest analytical facilities for centrifugation;
- Implementing the point-of-care (POCT) method in GP offices.

### 4.3. Feedback

In cases of doubt regarding the obtained result, the possibility of contacting the person who collected the sample or the laboratory staff to clarify procedural details.

## Figures and Tables

**Table 1:** Information regarding the collection of blood samples and the results of potassium testing.

Date	25 June	26 June
Time of blood sample collection	6.45 a.m.	11.30 a.m.
Time of collection of blood samples from the GP office by the courier van service	~1.00 p.m.	~1.00 p.m.
Result release time from the laboratory	4.25 p.m.	4.05 p.m.
Potassium [mmol/L]	6.4	5.1
Potassium reference values [mmol/L]	3.5–5.5	
Laboratory comment on the result	Repeated examination. Medical consultation is recommended.	-

**Table 2:** Reported cases of pseudohyperkalemia associated with blood sample storage conditions.

Author, Year	Patient	Potassium Test Result [mmol/L]	Repeated Potassium Test Result [mmol/L]	Presumed Cause of Pseudohyperkalemia
Hatano T. et al., 2025 [19]	22-year-old woman	6.6 mmol/L	4.0 mmol/L	Storage of a blood sample at low temperature in a patient with myotonic dystrophy type 1
Kuwano K. et al., 2023 [20]	73-year-old woman	8.0 mmol/L	3.4 mmol/L	Long-term storage/transport of a blood sample at low temperature in a patient with suspected familial pseudohyperkalemia
Kuwano K. et al., 2023 [20]	75-year-old woman	6.5 mmol/L	4.1 mmol/L	Long-term storage/transport of a blood sample at low temperature in a patient with suspected familial pseudohyperkalemia
Perez CC et al., 2022 [21]	57-year-old woman	a four-year history of mild hyperkalemia: 5.3–5.9 mmol/L in primary care	4.0 mmol/L (at the hospital)	Long-term storage/transport of a blood sample at low temperature in a patient with suspected familial pseudohyperkalemia
Smelli WS., 2007 [22]	72-year-old man	7.2 mmol/L	4.8 mmol/L	Long-term transport of a blood sample from GP office to laboratory (28 miles) by the courier van in a metal box under cold winter condition (ambient temperature −3 °C)
Takaki R. et al., 2025 [23]	70-year-old woman	multiple increased potassium results up to 9.7 mmol/L (when determined at the local clinic)	3.7 mmol/L (at the hospital)	Long-term storage/transport of a blood sample at low temperature in a patient with suspected familial pseudohyperkalemia
Xiong W. et al., 2022 [24]	56-year-old woman	multiple increased potassium results; 5.6–6.8 mmol/L (when determined at the local clinic)	4.3 mmol/L (at the hospital)	Storage of a blood sample at low temperature in a patient with familial pseudohyperkalemia associated with heterozygous mutation in the *ABCB6* gene

## Data Availability

The raw data supporting the conclusions of this article will be made available by the authors on request.

## Abbreviations

The following abbreviations are used in this manuscript:

ACE	angiotensin-converting enzyme
ISE	ion-selective electrode
GP	general practitioner
POCT	point-of-care
